# Work addiction and personality: A meta-analytic study

**DOI:** 10.1556/2006.2020.00097

**Published:** 2020-12-24

**Authors:** Bernadette Kun, Zsofia K. Takacs, Mara J. Richman, Mark D. Griffiths, Zsolt Demetrovics

**Affiliations:** 1 ELTE Eötvös Loránd University, Institute of Psychology, Budapest, Hungary; 2 ELTE Eötvös Loránd Universtity, Institute of Education, Budapest, Hungary; 3 Endeavor Psychology, Boston, MA, USA; 4 Psychology Department, Nottingham Trent University, Nottingham, United Kingdom

**Keywords:** work addiction, workaholism, personality, meta-analysis, Big Five personality traits

## Abstract

**Background:**

During the past three decades, research interest in work addiction has increased significantly. Most definitions concerning work addiction have specifically contained personality-related elements. However, the results of empirical studies concerning personality and work addiction are both few and mixed. The aim of the present study was to explore the role of personality in the background of work addiction.

**Methods:**

The present study systematically reviewed and empirically carried out a meta-analysis on all the published studies examining the association between personality variables and work addiction (*n* = 28).

**Results:**

The results of the meta-analysis indicated that perfectionism, global and performance-based self-esteem, and negative affect had the strongest and most robust associations as personality risk factors of work addiction. Among the Big Five traits, extraversion, conscientiousness, and intellect/imaginations showed positive relationships with work addiction. However, these associations were weak.

**Conclusions:**

Based on the meta-analysis, personality appears to explain only a small amount of the variance of work addiction and further studies are needed to assess the interaction between individual and environmental factors.

## Introduction

During the past few decades, research into behavioral addictions has increased significantly. While there are some behavioral addictions where some consensus has been reached about their conceptual validity (e.g., gambling disorder, and more recently, gaming disorder [Rumpf et al., 2018]), there are other types of behavioral addiction which still need further empirical validation in order to be accepted (Billieux, Schimmenti, Khazaal, Maurage, & Heeren, 2015; Petry, Zajac, & Ginley, 2018). Although “work addiction” is not included in either the DSM-5 (American Psychiatric Association, 2013) or the ICD-11 (Iler et al., 2019; World Health Organization, 2018), it has often been cited and referred to in the behavioral addiction literature. Oates published the first academic paper on “workaholism” in 1968 and his very frequently cited book (Oates, 1971) formed the basis for further research examining work addiction. Between 1968 and 2000, empirical and theoretical papers on work addiction sporadically appeared in the psychological literature. However, since the beginning of the millennium, scientific interest in work addiction has greatly increased. Given that individuals typically spend 8 h a day working which can lead to many gratifications in the workplace (e.g., salary, bonuses, health insurance, peer appreciation, and compliments), such reinforcements can sometimes lead to overwork. Although at first sight, work addiction might appear to be a positive behavior (Griffiths, Demetrovics, & Atroszko, 2018), it also has several adverse consequences to an individual's life and their personal environment. These negative (physical, psychological, and social) consequences (e.g., Andreassen, 2014; Andreassen & Pallesen, 2016; Quinones & Griffiths, 2015) highlight the need for further research into work addiction.

### Work addiction

There have been several attempts to define work addiction (see review by Clark, Michel, Zhdanova, Pui, & Baltes, 2016; Atroszko, Demetrovics, & Griffiths, 2019). In many studies, the terms “workaholism” and “work addiction” have been used interchangeably. However, these constructs are not the same. According to a recent review by Griffiths et al. (2018), “workaholism” is a more generic term that denotes anything related to high involvement in work and this term often incorporates several positive aspects of overwork (benefits, rewards, and respect). Contrarily, the term “work addiction” emphasizes the maladaptive patterns and the negative consequences of overwork in an addiction framework. Even so, some early authors used the term “workaholism” and even they highlighted the addiction-related symptoms of the problem. For example, Oates (1971) used (and developed) the term “workaholism” by describing a workaholic as a “*person whose need for work has become so excessive that it creates noticeable disturbance or interference with his bodily health, personal happiness, and interpersonal relations, and with his smooth social functioning*” (p. 4). Emphasizing the adverse consequences of excessive work has been a core element of other operational definitions of work addiction. Porter (1996) indicated that in work addiction there is an “*excessive involvement with work evidenced by neglect in other areas of life*”; p. 71). In the definition by Scott, Moore, and Miceli (1997), two components of addiction (i.e. salience and conflict; Griffiths, 2005) can be also found, in that individuals characterized by work addiction are “*those who spend a good deal of time in work activities at the expense of family and other outside obligations, who persistently think about work when they are not at work*” (p.292).

Conflicts with other areas of life as a negative consequence of work addiction were also described byRobinson (1998). He emphasized the loss of control in regulating work-related behaviors and the obsessive patterns of the problem. Therefore, Robinson (1998) articulated the addiction specific patterns of excessive work and also used the term “work addiction”. He defined work addiction as “*an obsessive-compulsive disorder that manifests itself through self-imposed demands, an inability to regulate work habits, and an overindulgence in work to the exclusion of most other life activities*” (p. 7) The lack of control over working is also an important element in the definition by Sussman (2012) who also highlighted the importance of negative consequences of work addiction.

More recently, work addiction has been conceptualized as a behavioral addiction. Based on the addiction components model (Griffiths, 2005), six core components of addictions are applicable for work addiction, namely (i) *salience* (work is the most important activity in the individual's life and controls her/his feelings, thoughts, and actions); (ii) *mood modification* (work is used as a coping strategy with negative mood states, and stress etc.); (iii) *tolerance* (increased amounts of work are necessary to experience the same psychological effects as previously); (iv) *withdrawal symptoms* (unpleasant mental and/or physiological effects when the individual is prevented from working); (v) *conflict* (the excessive amount of work generates conflict with other activities, important others, and oneself); and (vi) *relapse* (a tendency to return to the previous pattern of work after a period of controlled behavior or abstinence) (Griffiths, 2005; Griffiths & Karanika-Murray, 2012).

At the same time, some definitions represent more the concept of “workaholism”. Machlowitz (1980) suggested that the most important element of workaholism is the hard and intrinsic desire for work. Spence and Robbins (1992) defined a workaholic individual as someone who “*is highly work involved, feels compelled or driven to work because of inner pressures, and is low in enjoyment of work”* (p.162). In some other definitions, the enjoyment of work is also represented, while the negative aspects of overwork are narrowly articulated. Ng, Sorensen and Feldman (2007) consider individuals as workaholics if they *“enjoy the act of working, who are obsessed with working, and who devote long hours and personal time to work”* (p.114). Based on the conceptual differentiation between “workaholism” and “work addiction” by Griffiths and colleagues (2018), we interpret the models of Machlowitz (1980), Spence and Robbins (1992), and Ng et al. (2007) as theories of ’workaholism’ rather than “work addiction” since they do not emphasize the maladaptive patterns of overwork. This conceptualization is also true for the definition by Mudrack and Naughton (2001) which only highlights the considerable time and engagement in work and the influence and control of other people's work.

Based on the aforementioned (and other) definitions, several psychometric measurement instruments that assess work addiction and workaholism have been developed. Although these different concepts have frequently been used interchangeably, it is important to differentiate between measures of workaholism and work addiction. In Table 1, we summarize all the psychometric instruments currently available in the field of work addiction suggesting a possible categorization based on these two different terms.

**Table 1.** Psychometric measurement instruments in the field of work addiction

Measure	Authors	Key components of the concept	Term
Workaholism Battery (WorkBat)	Spence & Robbins (1992)	‘Workaholics’ are highly work involved, feels driven to work, and does not enjoy work	Workaholism
Work Addiction Risk Test (WART)	Robinson, Post & Khakee (1992)	Obsessive patterns in work, loss of control in regulating work habits, conflicts with other areas of life	Work addiction
Workaholism scale of the Schedule for Adaptive and Nonadaptive Personality (SNAP)	Clark (1996)	Does not feature any theoretical concept of work addiction	Workaholism
Non-required Work and Control of Others	Mudrack & Naughton (2001)	Excessive work which is not based on external requirements or economic situation and control of other people's work habits	Workaholism
Dutch Work Addiction Scale (DUWAS)	Schaufeli, Taris, & Bakker (2008)	Excessive work which is not based on external requirements, obsessive patterns in work, conflict with other areas of life	Work addiction
Bergen Work Addiction Scale (BWAS)	Andreassen, Griffiths, Hetland & Pallesen (2012)	Salience of work, mood modification by work, increased amounts of work necessary, withdrawal symptoms when the person is not allowed to work, conflicts because of excessive work, and relapse to overwork	Work addiction
Workaholism Analysis Questionnaire (WAQ)	Aziz, Uhrich, Wuensch & Swords (2013)	Excessive work, obsessive patterns in work, conflicts with other areas of life, withdrawal symptoms when the person is not allowed to work	Work addiction

In addition to emphasizing the adverse consequences of excessive work, in operational definitions of work addiction, internal and personality-related components are frequently described. Porter (1996) highlighted the *internal motives* of work rather than the characteristics of the job or organization. Other definitions have specifically contained personality-related elements, such as obsessive-compulsiveness. For instance, Robinson (1998a, 1998b) defined work addiction as an *obsessive-compulsive* disorder when overworking derives from poor self-worth, fear of failure, and anxiety. Similarly, Schaufeli et al. (2008) and Aziz, Uhrich, Wuensch and Swords (2013) also emphasized the individual's *obsession with working* in their definition.

These definitions suggest that personality plays an important role in work addiction. Based on these models, many studies have assessed the relationship between the risk of work addiction and different types of personality traits. The aim of such approaches is to explore the importance of personality in work addiction and identify the potential risk factors. As Griffiths et al. (2018) pointed out, the results of the studies on personality and work addictions are mixed. It appears that some personality factors show stronger relationships with work addiction, while others have a more questionable role (Atroszko, Andreassen, Griffiths, & Pallesen, 2016a, 2016b; Aziz, Zamary, & Wuensch, 2018; Falco, Piccirelli, Girardi, Di Sipio, & De Carlo, 2014; Spurk, Hirschi, & Kauffeld, 2015). To disentangle the mixed findings in the literature, the aim of the present paper was to systematically review the literature concerning work addiction and personality. Based on the differentiation between the two concepts of “workaholism” and “work addiction” described above, the present paper only focuses on those studies which were executed utilizing the concept of work addiction.

As Griffiths and colleagues (2018) emphasized, there are several individual, situational, and structural factors that contribute to work addiction. As is true for different addictive disorders, work addiction is based on an interaction between these different components, including (but not limited to) biological predispositions, social factors, the physical environment, and personality factors (Griffiths, 2005). Regarding work addiction, structural elements of work (e.g., number of hours working per day, financial rewards, the type or the familiarity of work, etc.) can also influence the risk of work addiction (Griffiths, 2011; Griffiths & Karanika-Murray, 2012). At the same time, the situational factors of work, such as social relationships at the workplace, the milieu of the workplace, and organizational culture and policies, can also contribute to work addiction (Griffiths, 2011). Although such factors have an impact on work addiction that exceed personality dimensions alone, the present paper only focuses on personality. Based on the existing literature in the field of work addiction, the present paper investigates and summarizes the individual contribution of personality traits to work addiction. The goal of the study is to empirically test the myth posited by Griffiths and colleagues (2018) that “work addiction exclusively occurs as a consequence of individual personality factors” (p. 4).

### Personality

Personality has been defined as *“psychological qualities that contribute to an individual's enduring and distinctive patterns of thinking, feeling and behaving”* (Cervone & Pervin, 2009, p. 8). Several models of personality have been developed by different theorists representing different perspectives. Among the dispositional perspective of personality, the Five Factor Model (FFM), more generally referred to as the “Big Five” model of personality (De Raad, 2000; Digman, 1990; Goldberg, 1993), has become the most widely accepted and well-known theory among personality psychologists. A large number of international studies have shown personality comprises five broad factors: *agreeableness, extraversion, conscientiousness, neuroticism* (vs. emotional stability), and *openness to experience* (or intellect, imagination, or culture). Although there is a general consensus concerning the FFM, there are other alternative comprehensive models of personality traits, such as the Temperament and Character Model (TCM, Cloninger, Svrakic, & Przybeck 1993). Cloninger and colleagues (1993) proposed a psychobiological model including four temperament dimensions (*novelty seeking, harm avoidance, reward dependence, and persistence*), and three dimensions of character (*self-directedness, self-transcendence, *and* cooperativeness*). Although the theoretical backgrounds of these models are different (namely, lexical hypothesis vs. biological factors), the dimensions of FFM and TCM have similarities and overlaps (De Fruyt, De Wiele, & Van Heeringen, 2000). Another alternative model for the big personality traits is the Big Two model (Digman, 1997). This model suggests that the big five traits can be combined into two higher-order factors, namely Alpha or Stability, and Beta or Plasticity. Alpha contains agreeableness, conscientiousness*,* and emotional stability, while Beta comprises extraversion and openness to experience (or intellect, imagination, or culture). In the present study, we include all the personality factors representing these models.

In addition to these aforementioned big personality models and the main personality traits these models comprise, there are other relevant personality dimensions that have to be considered in clinical and health psychology. In some cases, these other personality dimensions can be found in lower-order dimensions of the FFM or TCM but it can be very informative if they are also considered individually. For instance, *negative affectivity* (i.e., the tendency to feel negative emotions such as anger, anxiety, and guilt [Watson & Clark, 1984]) and *positive affectivity* (the tendency to feel positive emotions such as happiness, energy, and enthusiasm [Watson & Naragon, 2009]) relate strongly to the neuroticism and agreeableness factors of the FFM. However, these dimensions have further information that are additional to the big five traits (Watson, Wiese, Vaidya, & Tellegen, 1999). It is also true for other personality dimensions that are relevant to consider alone, such as obsessiveness, impulsiveness, trait anxiety, and narcissism. In the present study, we also include these personality dimensions which represent lower hierarchical levels of the main personality models.

Self-concept relates to an overall idea that individuals have about who they are. As Baumeister (1999) defines it, self-concept is* “the individual's belief about himself or herself, including the person's attributes and who and what the self is”.* Several associated terms can be interpreted within the self-concept model, such as “self-esteem” (i.e., the extent to which individuals like, accept, or approve of themselves, or how the individuals' value themselves), self-efficacy (i.e., individuals' judgments of their abilities), self-image (i.e., how individuals perceive themselves, a mental picture of themselves), ideal-self (i.e., what individuals wish they were really like), and several other aspects of the self. Perfectionism – a personality dimension characterized by *“high standards of performance which are accompanied by tendencies for overly critical evaluations of one's own behavior” *(Frost, Marten, Lahart, & Rosenblate, 1990, p. 450) can also be viewed as relating to the self-concept because perfectionist individuals' ideal-self is excellent and superb, therefore their self-image always has to improve itself.

It is also important to note that there are several aspects of self-esteem. Global self-esteem refers to an individual's overall sense of worthiness as an individual (Rosenberg, 1979). However, other important aspects of self-esteem, such as contingent self-esteem have been defined. Contingent self-esteem indicates the degree to which self-esteem is contingent upon achievements and outcomes (Kernis, 2002). Therefore, contingent self-esteem relates to performance-based self-esteem because a higher level of contingent self-esteem relates to more preoccupation with one's performance and evaluations. The personality factors representing self-concept were also included in the present study.

### Personality and work addiction

Personality risk factors for substance use disorders and behavioral addictions have been the focus of research for many decades. The existing literature on both chemical and behavioral addictions have demonstrated the importance of personality underlying these mental disorders and problematic behaviors (e.g., Grant, Potenza, Weinstein, & Gorelick, 2010; Stautz & Cooper, 2013). In the field of personality, a significant amount of research has examined the role of the big five personality traits in addictive disorders (e.g., Kotov, Gamez, Schmidt, & Watson, 2010). This has also been investigated in work addiction studies and many studies have also investigated the possible role of personality factors in maladaptive working patterns. More specifically, in several studies, a higher level of extraversion, neuroticism, and conscientiousness have been assumed to be personality risk factors of work addiction (e.g., Andreassen, Hetland, & Pallesen, 2010; Burke, Matthiesen, & Pallesen, 2006). However, “workaholism” and “work addiction” concepts have been used interchangeably in these studies, and therefore the present paper attempts to clarify the associations between big five traits and work addiction specifically. Considering the behavioral addiction concept of the term, it was expected that individuals working in an obsessive and maladaptive way, show higher levels of conscientiousness and neuroticism. At the same time, it seems logical that such individuals, who are more sensitive to rewards (and especially social rewards and recognition) try to do their best to have good relationships with others at their workplace (Van der Linden, Beckers, & Taris, 2007), and therefore show higher levels of extraversion (Depue & Collins, 1999; Gray, 1970). Regarding the aforementioned Temperament and Character Model (Cloninger et al., 1993), the studies examining the associations between these dimensions and work addiction are more limited (Di Nicola et al., 2010). The present study also included available studies investigating these personality traits regarding work addiction.

Emotional instability, difficulties in emotional regulation, and other emotional processes are also well documented in the topic of addictive disorders (Estévez, Jáuregui, Sánchez, Lopez-Gonzalez, & Griffiths, 2017; Siegel, 2015; Wilens, Martelon, Anderson, Shelley-Abrahamson, & Biederman, 2013). Regarding personality traits, trait negative affectivity has been emphasized as a possible risk factor in both substance use disorders and behavioral addictions. Negative affectivity has also frequently been assumed as an individual risk factor of work addiction (e.g., Ng et al., 2007; Scott et al., 1997). On the other hand, some theories (e.g., Killinger, 1991; Machlowitz, 1980; Spence & Robbins, 1992) which closely relate to the concept of “workaholism” have differentiated between positive and negative forms of the problem. As such, they propose that some “workaholic” individuals are characterized by positive affectivity. Consequently, it is important to clarify the associations of negative and positive affectivity with work addiction. The present study expected that “positive affectivity” would relate more to the concept “workaholism” and individuals characterized by “work addiction” would experience more negative affectivity.

Since the early 1980s, theories of work addiction have identified self-esteem, self-efficacy, and perfectionism as important correlates. However, these theories were based on observations and anecdotes only (e.g., Killinger, 1991; Machlowitz, 1980). Since 2000, empirical studies have begun to test the relationship between these constructs. Although several studies have reported a relationship between low levels of self-esteem, low self-efficacy, and work addiction, the results are mixed (e.g., Aziz et al., 2018; Burke & Matthiesen, 2004; Salmela-Aro & Nurmi, 2007). In a few studies, perfectionism – a personality dimension characterized by *“high standards of performance which are accompanied by tendencies for overly critical evaluations of one's own behavior”* (Frost, Marten, Lahart, & Rosenblatel, 1990, p.450) – has also been examined. According to these studies (e.g., Falco et al., 2017, 2014; Girardi et al., 2015; Stoeber, Davis, & Townley, 2013), although self-oriented perfectionism appears to be the most important type of perfectionism in work addiction, results on other-oriented perfectionism and socially prescribed perfectionism are mixed.

Based on the existing work addiction theories (e.g., Robinson, 1998; Porter, 1996), the present study expected that a higher level of work addiction would relate to lower levels of global self-esteem, self-efficacy, and a higher level of perfectionism. Regarding performance-based self-esteem, it seems logical to assume that those individuals who get more positive feedback from others concerning their achievement and quality of work, will think that they are more successful and valuable individuals. Therefore, those individuals who have a higher level of performance-based self-esteem and evaluate themselves only by their own performance and its appreciation by others may have a tendency to work more intensively and show the symptoms of work addiction.

In addition to the majority of the studies that have examined the big five traits or different aspects of the self-concept, other personality dimensions should also be considered in work addiction studies. Although several definitions of work addiction emphasize the importance of obsessiveness in work addiction (Robinson, 1998; Ng et al., 2007; Schaufeli et al., 2008), there is only one study that has investigated the association between these constructs (Butucescu & Uscatescu, 2013). Other personality dimensions, such as trait anxiety, type A personality, and narcissism have also been sporadically investigated in the field of work addiction (Clark, Lelchook, & Taylor, 2010; Robinson, 1999). In the present study, we also included these personality dimensions, although, the number of these studies was few. Based on the recurrent preoccupations of work characterized by individuals with work addiction, the present study expected that they would also have elevated levels of obsessiveness, trait anxiety, and type A behavior.

There are two previous meta-analytic studies on work addiction that have already examined the associations between personality factors and work addiction. However, in these meta-analyses, the authors did not take into account the conceptual differences between workaholism and work addiction. Patel, Bowler, Bowler and Methe (2012) conducted a meta-analysis, but they only examined studies using the Work Addiction Risk Test (WART, Robinson, 1998a; Robinson, Post & Khakee, 1992) and/or the Workaholism Battery (WorkBat, Spence & Robbins, 1992). As suggested above, the WART assesses work addiction, whilst the WorkBat assesses workaholism. Moreover, this meta-analysis was very limited because the authors included only two psychometric instruments, and their findings only comprised research that had been published before 2012. Patel et al. (2012) found a weak positive correlation between work addiction (as by the WART) and self-efficacy, and similarly, weak positive correlations between workaholism (as by WorkBat) and agreeableness, conscientiousness, extraversion, and self-efficacy. Additionally, they found a positive moderate relation with perfectionism.

In a later study, Clark and colleagues (2016) performed a meta-analysis on work addiction and focused on a number of correlates. However, the conceptual clarification of the terms “workaholism” and “work addiction” was also missing from this study and the authors conflated these concepts and included all the studies irrespective of the different psychometric measures of “workaholism” and “work addiction”. Among the dispositional variables they examined in their study, they identified perfectionism, negative affectivity, and Type A personality as significant moderate positive correlates of work addiction. Although extraversion was also correlated with work addiction, the relationship was very weak. Although Clark et al. (2016) and Patel et al. (2012) assessed many personality traits in relation to work addiction and workaholism, there are several reasons why a more up-to-date review and meta-analysis is now needed. Firstly, as noted above, we emphasize the importance of the differentiation between the concepts “workaholism” and “work addiction”. Based on the different definitions and models of the problem, we argue that “workaholism” and “work addiction” are not the same terms. Therefore, it is important to consider these conceptual differences in a meta-analysis. This clarification and the related possible differences were not considered in either of the two previous meta-analyses (i.e., Clark, Smith, & Haynes, 2020; Patel et al., 2012). Secondly, although the aim of the meta-analysis by Clark et al. (2016) was to include all the studies reporting a correlation between work addiction and one of the variables of their interest, the authors omitted seven relevant studies examining personality and work addiction (see details in the Methods section). Thirdly, the focus of the study by Patel and colleagues (2012) was very limited because it included only those studies using the WART and/or WorkBat. At the same time, in Clark et al.’s (2016) study, only specific dispositional variables were assessed as personality factors. These two earlier meta-analytic studies chose and analyzed perfectionism, type A personality, positive and negative affectivity, the big five factors, self-esteem, and self-efficacy. However, there are many other personality variables, such as narcissism, obsessive-compulsiveness, trait anxiety, and temperament personality dimensions which were not included in these studies. However, they had been investigated in relation to work addiction in earlier studies. Fourthly, their literature searches did not include any papers published since July 2013 (Clark et al., 2016) and before 2012 (Patel et al., 2012), respectively. Research into work addiction has increased significantly in recent years, and many studies have been published on work addiction in the past seven years (see details in the Method section). Fifthly, in these two previous meta-analytic studies, none of the possible moderator variables were tested in the relationship between personality and work addiction. However, these moderating factors may shed light on the inconsistencies among previous findings (e.g., self-esteem and work addiction, or perfectionism and work addiction). The present study proposed that the following moderator variables should be tested in a meta-analysis: age, gender, type of the sample (e.g., students vs. adult employees), and the instruments used to assess work addiction. If relevant, the possible differences between subscales of work addiction psychometric instruments should be also tested.

### The scope of the present study

In the present study, the aim was to synthesize all available empirical evidence regarding the relationship between any personality construct and work addiction. Based on the conceptual differentiation between “workaholism” and “work addiction” (Griffiths et al., 2018), the main scope of the present paper was to include only those studies investigated the term “work addiction”. Since the two existing meta-analytic studies did not make any distinction between these constructs, the current meta-analysis firstly summarizes the knowledge concerning the relationship between personality factors and work addiction interpreted in the addiction framework. We conducted a meta-analysis on all the studies using correlation analysis between any personality variable and work addiction. Compared to the previous meta-analytic studies, the present study (i) clearly differentiated between “workaholism” and “work addiction” and only included those studies which used the addiction framework, (ii) included relevant studies and personality factors that were missed in the previous meta-analyses, (iii) incorporated all the new relevant studies which have been published in the past seven years, and (iv) tested several moderator factors in the relationship between personality and work addiction, namely age, gender, study sample, and the psychometric measures of work addiction. Based on the literature and the previous meta-analytic results, the following hypotheses were considered for the meta-analysis:

**Hypothesis 1**

Among the big five traits, extraversion, conscientiousness, and neuroticism will correlate with work addiction.

1.a. There will be a positive correlation between extraversion and work addiction.
1.b. There will be a positive correlation between conscientiousness and work addiction.
1.c. There will be a positive correlation between neuroticism and work addiction.

**Hypothesis 2**

Among personality dimensions in the self-concept, self-efficacy and perfectionism will correlate with work addiction.

2.a. There will be a positive correlation between perfectionism and work addiction.
2.b. There will be a negative correlation between self-efficacy and work addiction

**Hypothesis 3**

Regarding self-esteem, it was assumed that global self-esteem and performance-based self-esteem will show a different direction to work addiction:

3.a. There will be a negative correlation between global self-esteem and work addiction.
3.b. There will be a positive correlation between performance-based self-esteem and work addiction.

**Hypothesis 4**

Based on the conceptual differentiation between “workaholism” and “work addiction” (Griffiths et al., 2018), it was assumed that “work addiction” will show different relationships with positive affectivity and negative affectivity.

4.a. There will be a positive correlation between work addiction and negative affectivity.
4.b. There will not be any significant correlation between positive affectivity and work addiction.

**Hypothesis 5**

Regarding the possible moderator variables, the following hypotheses were formulated:

5.a. Neither gender nor the mean age will have significant moderator effects on the relationship between personality and work addiction.
5.b. Work addiction is a problem that primarily affects adult individuals having a job. However, in several studies, work addiction has been investigated among college or university students (e.g., Bovornusvakool, et al., 2012; Clark et al., 2010). Overworking and outperforming in one's studies or in one's workplace might be different and may relate to dissimilar personality aspects. The structural and situational factors (Griffiths, 2011; Griffiths & Karanika-Murray, 2012) of a workplace and higher education are different and it may denote that personality factors give different contributions to the risk of work addiction. Therefore, we hypothesize that the type of the sample (namely, undergraduate students vs. working students. vs. adult employees) will have a moderating role between personality factors and work addiction.
5.c. Psychometric assessment of work addiction will not have a moderating effect on the relationship between personality factors and work addiction.

## Methods

The PRISMA-P (Preferred Reporting Items for Systematic Reviews and Meta-Analyses Protocols; Moher et al., 2009, 2015) were followed in the design, performance, and reporting of the present meta-analysis.

### Operational definitions

The aim of the present study was to examine the relationship between work addiction and personality. Studies that used the concept of “workaholism” were excluded. Therefore, all of the studies that assessed both work addiction and any personality variable were targeted. Studies were included if a specific psychometrically validated work addiction scale was used (see Table 1). The present study operationalized personality as referring to individual differences in characteristic patterns of thinking, feeling, and behaving (Kazdin, 2000). As for personality variables, the present study included biological (e.g., temperaments), dispositional (e.g., extraversion) traits, or aspects of the self-concept (e.g., self-esteem), which are part of the main personality psychology models.

### Search strategy

**Fig. 1.**
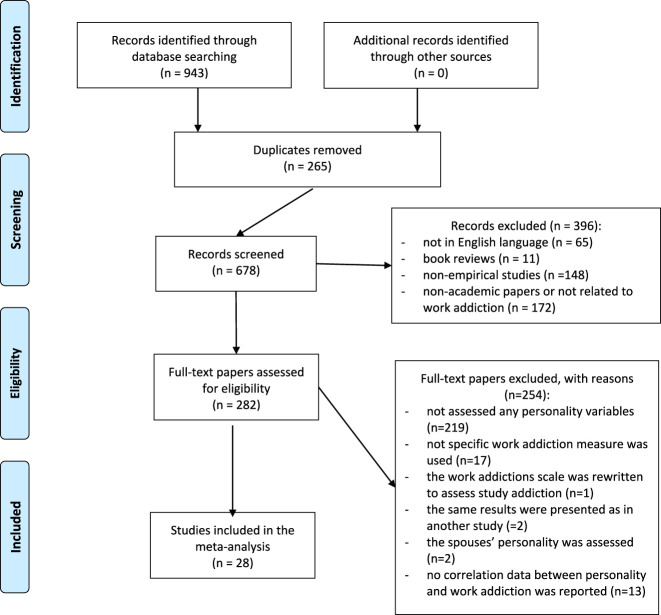
PRISMA flow diagram

For the present meta-analytic study, all the papers on work addiction were collected and coded into different fields of research (e.g., health psychology, organizational psychology, personality psychology, etc.). The papers were identified through a computerized literature search. The following databases were utilized: PsycINFO, PubMed, EBSCO, and SCOPUS. The search was performed to all the papers that were published before May 2020. Search terms included: “work addiction”, “work addict”, “workaholism” AND “workaholic”. Although the present study uses a clear distinction between “workaholism” and “work addiction”, in most primary studies these terms were used interchangeably, therefore it was necessary to search for both terms. As shown in Fig. 1, this procedure yielded 943 papers mentioning the topic of work addiction. The electronic search was supplemented by a manual search by reviewing the reference lists of the included studies in order to complement the database with further studies that were not found during the electronic database search. Additionally, a manual review of each paper was performed utilizing cross-references from original papers and reviews. No specific keywords for personality were used. Instead, all of the studies on work addiction were read to see if they included any personality variables or not.

### Inclusion and exclusion criteria

In the present review, studies were considered for inclusion if they provided empirical data on the relationship between work addiction and personality and had been published in English peer-reviewed journals or scientific books. Studies were included in the present study if they met the following criteria: (i) at least one work addiction scale was used; (ii) at least one scale or subscale was used to assess a personality variable; and (iii) the study was written in English language. The review focused only on work addiction, therefore other related studies that have investigated sub-types or precursors of work addiction such as “entrepreneurial addiction”, “study addiction”, and “studyholism” were not included for further analysis.

As shown in Fig. 1, studies were excluded if they were not in English language (*n* = 65), were doctoral dissertations (*n* = 21), were book reviews (*n* = 11), or were non-academic papers, or not related to work addiction (or irrelevant) (*n* = 172). All duplicates of the records from the different databases (*n* = 265) were also excluded. Two papers were excluded because they reported on the same data as studies already included in the meta-analysis. In addition, two other studies were also excluded because the risk of work addiction was only assessed in the context of the spouse's personality. One study was also excluded because the work addiction scale was reworded to an academic context for assessing study addiction. Following this procedure, 58 studies remained. Another inclusion criterion for the meta-analysis was that the study had to report a correlational coefficient regarding the relationship between work addiction and any of the personality variables. Therefore, studies were excluded that did not report a raw correlational coefficient between personality and work addiction, only the results of regression or path analysis (*n* = 13). Finally, studies were excluded if they used a psychometric instrument assessing the concept of “workaholism” (e.g., Workaholism Battery, Nonrequired Work and Control of Others, the Workaholism scale of the SNAP) (*n* = 17). Consequently, a total of 28 studies were included in the present meta-analysis (see Table 2).

**Table 2.** Summary of studies included the meta-analyses on the relationship between work addiction and personality

	Authors, year, country	Sample size	Population studied	Mean age (years)	% of males	Personality variables included in the meta-analysis	Work addiction scales	Scales used for personality factors
1	Andreassen, Bjorvatn, et al., 2016; Norway	1,267	Convenience sample of adult employees	35.7	9.8	Agreeableness,	BWAS	Mini-IPIP
						Extraversion,		
						Neuroticism, Conscientiousness, Openness		
2	Andreassen, Griffiths, et al., 2014; Norway	1,124	Nationally representative sample of adult employees	no data	49.0	Agreeableness, Extraversion, Neuroticism, Conscientiousness, Intellect/imagination	BWAS	Mini-IPIP
3	Atroszko et al., 2016a; Norway & Poland	1,560 + 1,264	Convenience samples of working students	30.2 + 24.5	30.6 + 22.2	Agreeableness,	BWAS	TIPI
						Extraversion,		
						Neuroticism, Conscientiousness. Openness		
4	Atroszko et al., 2017; Poland	723	Convenience sample of adult employees	36.4	27.7	Agreeableness,	BWAS	Mini-IPIP
						Extraversion,		
						Neuroticism, Conscientiousness, Openness		
5	Aziz, Zamary & Wuensch, 2018; USA	414	Convenience sample of adult employees	45.2	33.7	Self-esteem	WAQ	RSES
6	Bovornusvakool et al., 2012; USA	325	Convenience sample of undergraduate students	22.4	21.5	Positive affectivity, Negative affectivity, Perfectionism	WART	PANAS APS-R
7	Butucescu & Uscatescu, 2013; Romania	137	Convenience sample of adult employees	No data	46.7	Obsessive-compulsiveness, Trait anxiety	DUWAS	WABI
8	Clark, Lelchook & Taylor, 2010; USA	323	Convenience sample of working students	24.0	27.0	Perfectionism,	WART	APS-R,
						Positive affectivity		PANAS,
						Negative affectivity,		NPI,
						Narcissism, Agreeableness,		NEO-IPIP
						Extraversion, Neuroticism, Openness, Conscientiousness		
9	Clark, Smith, & Haynes, 2020; USA	661 + 150	Convenience samples of adult employees	35.1 + 38.5	54.0 + 33.0	Negative affectivity,	WART, DUWAS	PANAS, APS-R
						Perfectionism		
10	Di Nicola et al., 2010; Italy	158	Outpatients with bipolar disorder	48.7	41.0	Impulsiveness, Persistence, Novelty seeking, Harm avoidance, Reward dependence, Self-directedness, Cooperativeness, Self-transcendence	WART	BIS, TCI
11	Falco et al., 2017; Italy	770	Convenience sample of adult employees	no data	42.7	Perfectionism	DUWAS	HMPS
12	Girardi et al., 2015; Italy	413	Convenience sample of adult employees	no data	89.1	Perfectionism	DUWAS	HMPS
13	Gorgievski et al., 2014; Spain	180	Convenience sample of adult employees	42.0	59.1	Positive affectivity,	DUWAS	PANAS
						Negative affectivity		
14	Guglielmi et al., 2012; Italy	224	Convenience sample of adult employees	no data	33.0	Self-efficacy	DUWAS	NGSES
15	Kezdy et al., 2013; Hungary	215	No information	37.9	38.0	Self-esteem	WART	RSES
16	Laconi et al., 2015; France	378	Convenience sample of adult employees	24.4	21.0	self-esteem	BWAS	RSES
17	Mazzetti, Biolcati et al., 2016; Italy	269	Convenience sample of adult employees	47.4	55.8	Positive affectivity,	DUWAS	PANAS
						Negative affectivity		
18	Mazzetti, Chiesa, et al., 2016; Italy	295	Convenience sample of adult employees	35.1	24.6	Neuroticism	DUWAS	BFQ short
19	Mazzetti, Schaufeli & Gugliemi, 2014; Italy	333	Convenience sample of adult employees	45.4	51.4	Perfectionism, Conscientiousness, Self-efficacy,	DUWAS	BFI
								MPS self-efficacy scale by Mazzetti et al. (2014)
20	Pšeničny & Perat, 2016; Slovenia	3,393	Random sample of adult employees	36.0	27.5	Self-esteem	WART	PBSES
21	Quinones et al., 2016; United Kingdom	516	Convenience sample of adult employees	45.1	50.2	Agreeableness,	DUWAS	Mini-IPIP
						Extraversion,		
						Neuroticism, Conscientiousness,		
						Openness		
22	Robinson, 1999, USA	371	Convenience sample of undergraduate students	22.0	29.0	Type A behavior,	WART	JAS, TASRI,
						Trait anxiety		STAI
23	Spurk et al.2016; Germany	685	Convenience sample of adult employees	32.4	41.8	Agreeableness,	DUWAS	TIPI
						Extraversion,		
						Neuroticism, Conscientiousness,		
						Openness,		
24	Stoeber et al., 2013; United Kingdom	133	Convenience sample of adult and student employees	no data	16.5	Perfectionism	DUWAS	MPS
25	Taris et al., 2010; The Netherlands	199	Convenience sample of adult employees	39.6	58.8	Perfectionism	WART	FMPS
26	van Wijhe, Peeters, Schaufeli, & van den Hout, 2011; The Netherlands	173	Convenience sample of adult employees	38.4	48.0	Positive affectivity,	DUWAS	PANAS
						Negative affectivity		
27	van Wijhe, Peeters, & Schaufeli, 2014; The Netherlands	191	Convenience sample of adult employees	39.1	34.6	Self-esteem	DUWAS	PBSES
28	Wojdylo et al., 2013; Poland	1,459	Convenience sample of adult employees	38.8	40.9	Self-esteem	WART	RSES

*Note*: WART = Work Addiction Risk Test; BWAS = Bergen Work Addiction Scale; DUWAS = Dutch Work Addiction Scale; WAQ = Workaholism Analysis Questionnaire NEO-IPIP = International Personality Item Pool Representation of the NEO PI-R; TIPI = Ten Item Personality Inventory; Mini-IPIP = Short form of the International Personality Item Pool Representation; BFQ short = Big Five Questionnaire short form; BFI = Big Five Inventory; PANAS = Positive and Negative Affect Schedule; RSES = Rosenberg Self-Esteem Scale, APS-R = Almost Perfect Scale Revised; NGSES = New General Self-Efficacy Scale; MPS = Multidimensional Perfectionism Scale; FMPS = Frost Multidimensional Perfectionism Scale; HMPS = Hewitt Multidimensional Perfectionism Scale; PBSES = Performance-Based Self-Esteem Scale; WABI = Work Attitudes and Behaviors Inventory; BIS = Barratt Impulsiveness Scale; TCI = Temperament and Character Inventory (TCI) – Revised Version; JAS = Jenkins Activity Survey; TASRI = Type A Self-Report Inventory; STAI = State-Trait Anxiety Inventory; NPI = Narcissistic Personality Inventory.

Comparing the two earlier meta-analyses on work addiction, Clark et al. (2016) included only 16 studies on personality and work addiction (and workaholism) and Patel et al. (2012) included only nine studies on these topics. As aforementioned, several relevant studies on personality were overlooked in the comprehensive meta-analysis by Clark and colleagues (2016). Namely, an additional seven studies investigated work addiction and any personality variables (Bovornusvakool, Vodanovich, Ariyabuddhiphongs, & Ngamake, 2012; Butucescu & Uscatescu, 2013; Di Nicola et al., 2010; Kezdy, Martos, & Robu, 2013; Stoeber et al., 2013; Taris, van Beek, & Schaufeli, 2010; Wojdylo, Baumann, Buczny, Owens, & Kuhl, 2013) were not reported in that study. Moreover, our meta-analysis comprised a further 17 studies that have been published since Clark and colleagues' (2016) paper was published. However, we excluded eight studies from the previous meta-analysis based on theoretical reasons (i.e., the term “workaholism” was assessed). Consequently, 24 studies (85.7%) in the total included 28 studies in the present meta-analysis were not synthesized in any of the two previous meta-analyses.

### Coding

Every paper was coded according to a predefined coding schema regarding the following information: (i) bibliographic information (title, author(s), year of publication, and the country where the data were collected); (ii) sampling method (convenience, systematic, or representative sampling); (iii) sample characteristics (the number and the mean age of the participants in the study; the percentage of males in the study); (iv) personality variables assessed in the study (e.g., self-esteem, extraversion, etc.); (v) the kind of personality measure used in the study (e.g., Rosenberg Self-Esteem Scale, Ten Item Personality Inventory, etc.); and (vi) the kind of work addiction instrument used in the study (e.g., Work Addiction Risk Test, Bergen Work Addiction Scale, etc.).

### Statistical analyses for meta-analysis

The present meta-analysis was conducted with Comprehensive Meta-Analysis, Version 2.0 software (Borenstein et al., 2005). The meta-analysis used the correlations and sample sizes reported in the studies. If a study reported correlations with more than one personality variable, the software took the mean average of those before including it in meta-analytic estimates. The random-effects model was used to determine whether the average correlations were statistically significant. To test the homogeneity of the effect sizes across studies for each measure, the Cochran *Q*-statistic and the I-square statistic were used (Hedges & Olkin, 1985). The software weights the studies when calculating the average effect size so that studies with larger samples weight more in the average. Following this, the symmetry of the funnel plot was used to assess possible publication bias as well as the Begg and Mazumdar (1994) and Egger, Smith, Schneider and Minder (1997) publication bias tests. Additionally, Rosenthal's fail-safe number (Rosenthal, 1979) was calculated, which is an estimate of how many studies with non-significant results would turn the average effect non-significant. A fail-safe *n* exceeding 5*k*+10 is considered a robust effect.

In case of heterogeneous effects, potential categorical moderators were assessed with the *Q*-statistic: (i) different measures of work addiction (scales and subscales, and (ii) type of the sample (undergraduate students, working students, adult employees). Finally, meta-regression analyses were performed on two possible numerical moderator variables: the average age and the gender distribution of the samples.

## Results

### Description of the studies

As shown in Table 2, the 28 studies (30 independent effect sizes in 28 studies) included in the meta-analysis were published between 1999 and 2020. Most of the studies were conducted in Europe (82.1%), and five studies (17.9%) were conducted in North America (USA). All but one study used a convenience sample of participants (with the one non-convenience study being a Norwegian study a nationally representative sample of adult employees). In 23 studies (82.1%), the sample comprised adult employees (mean age from 24.4 to 47.4 years), in two studies undergraduate students were recruited (mean age from 22.0 to 22.4 years), in two studies the sample comprised students that were employed (mean age from 24.0 to 30.2 years) were included, and in one study outpatients with bipolar disorder were included (mean age of 41 years). The average sample size of the 28 studies was 653.7. The average ratio of males in the samples was 41.4%. The following work addiction assessment instruments were applied: Dutch Work Addiction Scale (DUWAS) in 14 studies, Bergen Work Addiction Scale (BWAS) in five studies, Work Addiction Risk Test (WART) in nine studies, and Workaholism Analysis Questionnaire (WAQ) in one study (although the name of the measure contains the word “workaholism”, the scale operationalizes several components of the concept of “work addiction”). The following personality variables were assessed in the 28 studies (the number of studies are in parentheses): neuroticism (*n* = 9), conscientiousness (*n* = 9), extraversion (*n* = 8), agreeableness (*n* = 7), openness (*n* = 6) intellect/imagination (*n* = 2), self-esteem (*n* = 5), self-efficacy (*n* = 2), perfectionism (*n* = 12), positive affectivity (*n* = 5), negative affectivity (*n* = 7), trait anxiety (*n* = 2), type A behavior (*n* = 1), obsessiveness (*n* = 1), impulsiveness (*n* = 1), narcissism (*n* = 1), harm avoidance (*n* = 1), reward dependence (*n* = 1), persistence (*n* = 1), novelty seeking (*n* = 1), cooperativeness (*n* = 1), self-directedness (*n* = 1), and self-transcendence (*n* = 1). Regarding these personality variables, we calculated the effects for these traits separately and also grouped them into three personality concepts: (i) Big Five traits, (ii) self-related personality traits (self-esteem, self-efficacy, self-transcendence, self-directedness, and perfectionism), and (iii) positive and negative affectivity traits. The average effect sizes for these groups of variables were based on the absolute value of correlation coefficients in the primary studies because we expected some to positively and some to negatively correlate with work addiction. In order to avoid these effects canceling each other out, we utilized the absolute values. There were no outliers (i.e., no studies with a standardized residual exceeding ±3.29).

### Main results of the meta-analysis

**Fig. 2.**
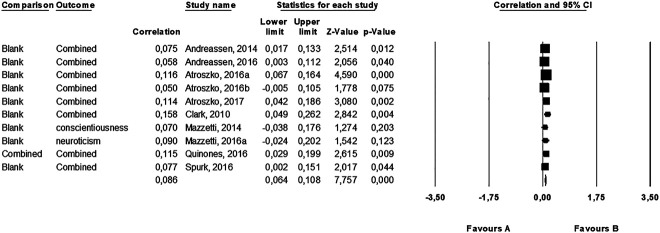
Forest plot of the studies in which we calculated an average correlation between Big Five traits and work addiction

Regarding the Big Five traits, we calculated the average effect size based on the absolute value of each correlation coefficients because (as aforementioned) we expected some traits to positively and others to negatively correlated with work addiction. We found a small average correlation in the studies (*r* = 0.10, *k* = 10, 95% CI [0.08, 0.13], *P* < 0.001) (Fig. 2). This effect was homogenous (*Q* [9] = 7.76, *P* = 0.558, *I*^2^ = 0.00). Analysis of publication bias revealed a symmetrical funnel plot. The fail-safe N showed that 201 “null” studies would need to be found and put in the analysis to negate the effect, which suggests a robust effect. Finally, neither the Begg's test (*P* = 0.788) nor the Egger's test (*P* = 0.947) was significant. Consequently, no evidence of publication bias was found. As shown in Table 3, none of the Big Five traits showed a moderate or strong relationship with work addiction. Only extraversion, conscientiousness, and intellect/imagination significantly correlated with work addiction, but the correlation coefficients were small (never higher than 0.104). Although our first hypothesis was confirmed because extraversion showed a significant positive correlation with work addiction, this correlation was negligible. Conscientiousness was also found to be a negligible factor of work addiction. However, the coefficient showed the opposite (negative) direction than we had expected. Finally, although intellect/imagination correlated with work addiction positively and significantly, the results are limited because only two studies assessed this personality variable. We also analyzed the main personality factors by using the Big Two concept (Digman, 1997). The Alpha/Stability factor (which contains agreeableness, conscientiousness, and neuroticism) showed a significant positive correlation with work addiction (*r* = 0.10, *k* = 10, 95% CI [0.07, 0.14], *P* < 0.001) and the Beta/Plasticity factor (comprising extraversion and openness to experience/intelligence) also correlated to work addiction positively (*r* = 0.06 *k* = 8, 95% CI [0.03, 0.09], *P* < 0.001) (see Table 3).

**Table 3.** Main result of the meta-analysis on the relationship between work addiction and personality

Outcome	*N*	*k*	*R*	CI 95%	*Z*	*P*
*Big Five traits*						
Extraversion	7,462	8	**0.040**	0.018; 0.063	3.483	<0.001
Neuroticism	7,757	9	0.115	−0.012; 0.238	1.780	0.075
Agreeableness	6,777	7	0.002	−0.057; 0.061	0.065	0.949
Conscientiousness	7,795	9	−**0.061**	−0.115; −0.006	−2.185	0.029
Openness	3,663	4	0.097	−0.001; 0.193	1.935	0.053
Intellect/imagination	3,114	3	**0.104**	0.055; 0.153	4.135	<0.001
Overall Big Five	8,090	10	**0.104**	0.082; 0.125	9.350	<0.001
Overall Stability/Alpha	8,090	10	**0.101**	0.065; 0.137	5.492	<0.001
Overall Plasticity/Beta	7,462	8	**0.061**	0.030; 0.092	3.823	<0.001
*Self-related personality factors*						
Self-esteem	5,185	6	−0.095	−0.307; 0.126	−0.838	0.402
Global self-esteem	1,187	4	−**0.257**	−0.350; −0.182	−6.539	<0.001
Performance-based self-esteem	3,584	2	**0.245**	0.054; 0.418	2.496	0.013
Self-efficacy	557	2	−0.036	−0.119; 0.047	−0.849	0.396
Perfectionism	2,645	12	**0.299**	0.235; 0.360	8.813	<0.001
Narcissism	323	1	**0.240**	0.143; 0.340	4.379	<0.001
Self-directedness	158	1	−0.140	−0.290; 0.017	−1.755	0.079
Self-transcendence	158	1	**0.180**	0.025; 0.327	2.266	0.023
Overall	8,213	16	**0.267**	0.206; 0.326	8.278	<0.001
*Positive and negative affectivity*						
Positive affectivity	1,270	5	−0.017	−0.072; 0.039	−0.593	0.553
Negative affectivity	2081	7	**0.321**	0.221; 0.414	6.053	<0.001
Overall	2081	7	**0.226**	0.160; 0.289	6.619	<0.001
*Other factors*						
Trait anxiety	508	2	**0.383**	0.306; 0.455	9.043	<0.001
Type A behavior	371	1	**0.437**	0.351; 0.516	8.994	<0.001
Obsessiveness	137	1	**0.380**	0.227; 0.515	4.631	<0.001
Impulsiveness	158	1	0.080	−0.077; 0.233	0.998	0.318
Harm avoidance	158	1	0.000	−0.156; 0.156	0.000	1.000
Reward dependence	158	1	−0.090	−0.243; 0.067	−1.124	0.261
Persistence	158	1	**0.440**	0.305; 0.558	5.879	<0.001
Novelty seeking	158	1	−0.050	−0.205; 0.107	−0.623	0.533
Cooperativeness	158	1	0.110	−0.047; 0.262	1.375	0.169

*Note*: Bold text indicates a statistically significant correlation with a *P*-value less than 0.05.

**Fig. 3.**
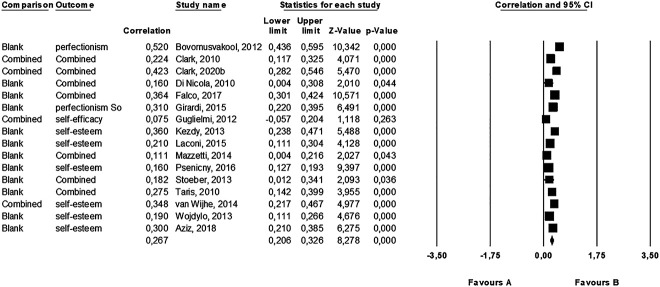
Forest plot of the studies in which we calculated an average correlation between personality variables that related to self-concept and work addiction

Again, we calculated an average effect size from the absolute value of each correlation coefficients for those personality variables that related to self-concept (self-esteem, self-efficacy, self-transcendence, self-directedness, and perfectionism) as we expected some to positively and some to negatively relate to work addiction. Overall there was a small average correlation in the studies (*r* = 0.27, *k* = 16, 95% CI [0.21, 0.33], *P* < 0.001) (see Table 3). This effect was heterogeneous (*Q* [16] = 105.46, *P* < 0.001, *I*^2^ = 85.78) (see Fig. 3 for the forest plot). Analysis of publication bias revealed a symmetric funnel plot. The fail-safe *N* showed that 1,782 “null” studies would need to be found and put in the analysis to negate the effect, which suggests a robust effect. Additionally, neither the Begg's test (*P* = 0.589) nor the Egger's test (*P* = 0.100) was significant. In sum, there was no evidence of publication bias. Analyzing these personality traits one-by-one (see Table 3), the most robust result found was the correlation between perfectionism and work addiction (*r* = 0.30; *P* < 0.001). The meta-analysis involving 16 studies showed that the higher the level of perfectionism, the higher the risk of work addiction. Although it was also found that both narcissism and self-transcendence related positively to work addiction, these results are not strongly reliable because only one study tested the possible relationship, respectively. Interestingly, the correlation between self-esteem and work addiction was not significant. However, we previously assumed that it would be necessary to differentiate between global self-esteem and performance-based self-esteem regarding work addiction. After we tested these constructs separately, it was confirmed that global self-esteem had a significant, negative, and weak correlation with the risk of work addiction, while performance-based self-esteem showed a significant positive and weak correlation with work addiction. Contrary to expectation, we did not find a significant correlation between self-efficacy and work addiction. However, the number of studies was very limited (*n* = 2).

**Fig. 4.**
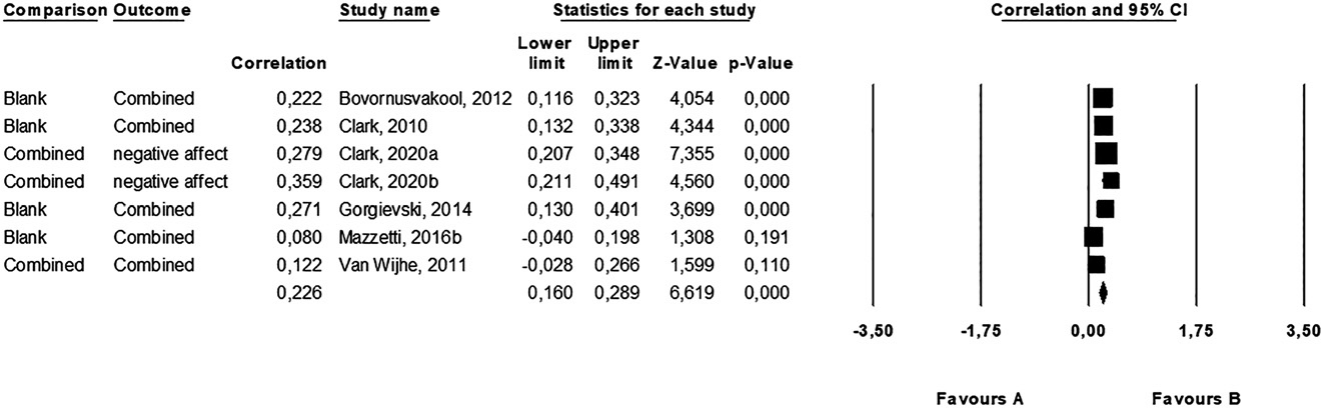
Forest plot of the studies in which we calculated an average correlation between positive affectivity, negative affectivity and work addiction

Regarding positive affectivity and negative affectivity, again, as we expected the first to negatively correlate with work addiction and the second to positively correlate with work addiction, we calculated an overall estimate using the absolute value of each correlation coefficients, and we found a small average correlation in the studies (*r* = 0.23, *k* = 7, 95% CI [0.16, 0.29], *P* < 0.001) (Table 3 and Fig. 4). This effect was heterogeneous (*Q* [6] = 13.585, *P* = 0.035, *I*^2^ = 55.83). Analysis of publication bias revealed a symmetric funnel plot. The fail-safe *N* revealed that 182 “null” studies would need to be found and put in the analysis to negate the effect, which suggests a robust effect. Additionally, neither the Begg's test (*P* = 0.880) nor the Egger's test (*P* = 0.661) was significant. In sum, we found no indication of publication bias. When considering the two forms of affectivity separately, only negative affectivity showed significant (positive and moderate) average correlation with work addiction (*r* = 0.32, *P* < 0.001), while positive affectivity was not related to it.

Among the other personality variables, we found that trait anxiety, type A behavior, obsessiveness, and persistence showed significant correlations with the risk of work addiction (Table 3). All of these factors correlated positively and moderately with work addiction. However, there were very few studies investigating the association between these factors and work addiction (two studies on trait anxiety, and only one-one study respectively on the other factors). None of the other personality factors, namely impulsiveness, harm avoidance, reward dependence, novelty-seeking, and cooperativeness, correlated with work addiction according to the results. Again, these results are based only on one-one study per personality variable (Table 3).

### Moderator factors

#### Age and gender

We assessed the effect of the average age and gender distribution of the sample. Six studies did not report the average age of the sample. In the rest of the studies, we tested the effect of average age for the three main groups of personality factors (Table 4). The average age was not a significant moderator between Big Five traits and work addiction, between self-related personality factors and work addiction, and between positive and negative affectivity and work addiction. We found the same results for the other moderator factor as well: gender distribution was also not a significant moderator between Big Five traits and work addiction, between self-related personality factors and work addiction, and between positive and negative affectivity and work addiction. In sum, our hypotheses regarding both age and gender were confirmed.

**Table 4.** Effects of the gender distribution and the mean age in the relationship between work addiction and personality factors

Moderator	Outcome	Coefficient	*Z*	*P*
Gender	Agreeableness	0.0022	0.93	0.3500
	Extraversion	0.0006	0.61	0.5445
	Neuroticism	0.0022	0.39	0.6997
	Conscientiousness	0.0021	1.00	0.3188
	Openness	0.0046	0.92	0.3595
	Big Five overall	−0.0001	−0.08	0.9377
	Self-related overall	−0.0040	−1.07	0.2854
	Perfectionism	−0.0059	−1.18	0.2371
	Positive and negative affectivity overall	−0.0015	−0.57	0.5660
	Negative affectivity	−0.0038	−1.01	0.3148
	Positive affectivity	−0.0013	−0.74	0.4589
Mean age	Agreeableness	0.0035	0.74	0.4565
	Extraversion	−0.0013	−0.62	0.5360
	Neuroticism	0.0185	1.61	0.1070
	Conscientiousness	0.0029	0.72	0.4687
	Openness	0.0048	0.75	0.4516
	Big Five overall	0.0012	0.64	0.5219
	Self-related overall	−0.0058	−1.27	0.2054
	Perfectionism	−0.0069	−0.81	0.4203
	Positive and negative affectivity overall	−0.0031	−0.74	0.4620
	Negative affectivity	−0.0086	−1.56	0.1196
	Positive affectivity	−0.0027	−0.99	0.3255

#### Type of the sample

We analyzed the effects according to the type of sample the primary studies applied: adult employees, undergraduate students, and working students (for all the details see the Supplementary data). As there were only a handful of studies in the different categories, we did not statistically contrast the effects in the different categories. Instead, we report the effects in each category descriptively. The only personality variables tested were those that had studied at least two different sample types. Therefore, we conducted moderator analysis only for the following personality factors: extraversion, agreeableness, neuroticism, conscientiousness, openness, perfectionism, negative affectivity, and positive affectivity. Among Big Five traits, we found that agreeableness did not relate to work addiction in any sample. However, we found some differences in the results based on the samples for extraversion, neuroticism, conscientiousness, and openness. Although extraversion showed a weak positive correlation with work addiction in working samples (adult employees and working students), the correlation was not significant among undergraduate students. We also found a similar pattern for openness. Regarding conscientiousness, we found the opposite tendency: while we found a significant negative, weak correlation with work addiction among undergraduate students, the correlation was not significant among either adult employees or working students. Finally, we found that while a higher level of neuroticism related to work addiction among adult employees, in contrast, there was a significant, negative, and moderate correlation between neuroticism and work addiction among working students (*r* = −0.44, *P* < 0.0001). However, it is important to note that only one study included a working student sample, therefore these results are not robust.

Regarding perfectionism, the direction of the association with work addiction was the same in all three types of samples but we found that this positive correlation appeared somewhat stronger among undergraduate students (*r* = 0.52, *P* < 0.0001) than among the two working samples (*r* = 0.32, *P* < 0.0001, and *r* = 0.12, *P* < 0.0001, respectively). In regards to positive affectivity, the different samples showed similar results, namely, this personality variable did not show significant correlations with work addiction in any of the three types of samples. However, regarding negative affectivity, moderate positive correlation coefficients were found among undergraduate and working students (*r* = 0.41, *P* < 0.0001) while in adult employees, the positive correlation was small (*r* = 0.29, *P* < 0.0001).

#### Psychometric measures of work addiction

We assumed that the measurement instrument of work addiction would not have a moderating effect on the relationship between personality factors and work addiction (for all the details see the Supplementary data). Again, because there were few studies in the different categories, we did not statistically contrast the effects. Almost similar results were found for the different work addiction instruments regarding self-esteem, perfectionism, positive affectivity, and negative affectivity. Therefore, the moderating effect of the operationalization of work addiction does not appear to be relevant in the relationship between these personality factors and work addiction. However, results for the Big Five traits were mixed. Regarding extraversion, although the correlations were always very weak, we found significant relationships only with the BWAS and the WART scales and the “Working Compulsively” subscale of the DUWAS scale. These scales represent the more problematic aspects of overwork, while the “Working Excessively” subscale, which did not correlate with extraversion, mainly assesses the extreme amount of work. Only the “Working Compulsively” subscale of DUWAS showed a significant and weak positive correlation with agreeableness, while none of the other scales correlated with this personality trait. However, this significant result was based only on a single study. We found mixed results regarding both conscientiousness and neuroticism. While there was a significant and negative correlation between conscientiousness and BWAS, the direction was the opposite with the DUWAS-10 scale and “Working Compulsively”. Again, for neuroticism, although BWAS and the subscales of the DUWAS correlated positively with neuroticism, the only study investigating the association between neuroticism and the WART showed a significant and moderate negative correlation. Finally, results concerning openness were mostly homogenous because all the instruments except BWAS showed significant, positive, and weak correlation with this personality trait.

## Discussion

A growing body of literature has focused on the possible risk factors of work addiction (Sussman, 2012). Due to mixed results in earlier studies, the significance of underlying personality factors of work addiction is still unclear. Therefore, the present meta-analysis examined the relationship between work addiction and personality. In the first half of the 2010s, two meta-analyses were conducted in the field of work addiction and both of them included personality factors in relationship to work addiction (Clark et al., 2016; Patel et al., 2012). However, we performed a more up-to-date meta-analysis based on several clear reasons: (i) we theoretically made a distinction between “workaholism” and “work addiction”, and we only included the studies assessing the concept of “work addiction”; (ii) several relevant studies on work addiction and personality were not included in the previous analyses; (iii) some of the relevant personality traits were not included in the previous meta-analyses; (iv) in the last six years, several new relevant studies have been published and we also included these studies; and (v) in the previous meta-analyses, the possible effect of different moderator variables (e.g. age, gender, type of the sample, and type of the work addiction measure) were not tested, and we also conducted these new analyses.

In total, 28 studies met the inclusion criteria, involving a wide range of personality variables. Although several studies already investigated the association between work addiction and the big five traits and found statistically significant relationships, our meta-analysis demonstrated the lack of a substantial associational role of the big five factors in work addiction.

In the earlier meta-analyses (Clark et al., 2016; Patel et al., 2012), the authors only included five studies examining the big five personality traits in the meta-analysis, including both the scales of “workaholism” and “work addiction”. The results of the present study demonstrated that work addiction showed the same associations between extraversion and work addiction, along with conscientiousness and intellect/imagination also being significantly correlated with work addiction. Therefore, our hypotheses regarding the big five traits were only partially supported. Earlier theories have characterized individuals with work addiction as persistently thinking about work, obsessed with their work, and have an increased need for control (Mudrack & Naughton, 2001; Robinson, 1998a; Scott et al., 1997), the results of the meta-analysis presented here showed that being more controlling, stubborn, focused, and less flexible and spontaneous (i.e., being more conscientious) were slight risk factors of work addiction.

We also expected that a higher level of extraversion would be a risk factor of work addiction because individuals characterized by work addiction have a higher need for social feedback from others about their achievement, abilities, and success (Van der Linden et al., 2007). Although the correlation coefficient was low, we found a significant positive correlation with work addiction and extraversion. At the same time, contrary to our assumptions, having more emotional instability (and therefore showing elevated emotional distress) was not related to the risk of work addiction. However, the novelty of the present study concerns the different moderator variables (such as the measures and subscales of work addiction, the type of the sample, and the effect of gender and age) in the relationship between work addiction and the big five traits.

Regarding neuroticism, we found different results based on the different psychometric instruments. Interestingly, while the Work Addiction Risk Test showed a moderate negative correlation with work addiction, all the other psychometric scales and subscales related positively to emotional instability. However, we should note that there was only one study using the WART (Clark et al., 2010), therefore this contradictory finding could be a methodological artefact. Although there is a moderate-high correlation between WART and the BWAS (Andreassen, Griffiths, Hetland, & Pallesen, 2012; Andreassen, Ursin, Eriksen, & Pallesen, 2012), this result might suggest that there are some conceptual differences between these scales which lead to different results regarding neuroticism. Another important result we found regarding neuroticism was the differences between the samples. Although neuroticism did not relate to work addiction among students, we found a higher level of neuroticism among adult employees with an elevated risk of work addiction. We also found the same difference regarding openness. It appears that this personality factor plays a significant role in underlying work addiction among adult employees. However, the level of openness among students was not relevant to their risk of work addiction. These aforementioned personality traits represent different aspects of the Big Two model (Digman, 1997), namely, extraversion and openness/intellect/imagination are components of the Beta/Plasticity superfactor whilst conscientiousness, neuroticism (and agreeableness) belong to the Alpha/Stability factor. Our results showed that both of these higher-order factors show the same (weak positive) associations with work addiction.

Conclusively, some of the main personality traits have specific effects on work addiction among employed adult people but not in another context (i.e., attending education) so the individual risk factor of personality appears unimportant. We assume that in this period, other social and situational factors (e.g., peer pressure and current social life) are also very important in the motivation of excessive work (Sunil & Jyoti, 2019). In the present authors' opinion, the validity of using work addiction scales among student samples is questionable. For these individuals, the problem of “study addiction” (which was defined byAndreassen, Hetland and Pallesen [2014, p. 8] as *“being overly concerned with studying, to be driven by an uncontrollable motivation to study, and investing so much energy and effort into studying that it impairs private relationships, spare-time activities, and/or health”*) could be more relevant. The results here are partially in line with studies on “study addiction” because Atroszko et al. (2016a, 2016b) found that study addiction in a Norwegian student sample did not correlate with openness, but extraversion showed a significant negative weak correlation with study addiction (as a possible antecedent of work addiction [Kun, 2018]).

Although several theories of work addiction have emphasized the importance of self-related aspects of personality (Fassel, 1992; Killinger, 1991; Machlowitz, 1980; Ng et al., 2007; Porter, 1996), the results of the present study confirmed that only perfectionism, narcissism, and self-transcendence showed a positive but weak relationship with work addiction. The analysis affirmed theories that posit perfectionism as an important personality factor underlying work addiction. For instance, Machlowitz (1980) defined work addiction as an “obsessive-perfectionism”, and Scott et al. (1997) proposed three different types of work addiction: compulsive-dependent, perfectionist, and achievement-oriented. Porter (2004) characterized “workaholics” as individuals who *“are prone to rigid thinking; they are not able to be flexible in their ideas. This results in perfectionist attitudes that exceed simple maintenance of high standards” *(p. 435). These hypotheses were supported because the meta-analysis demonstrated a significant positive relationship between perfectionism and work addiction. When considering the different work addiction instruments, the present study found that all the psychometric measures of work addiction showed a significant positive correlation with perfectionism, therefore this result was robust. Although correlation analysis is not suitable for explaining any causal relationship, the present authors assume that a higher level of perfectionism may be an individual antecedent of work addiction. If individuals always want to be better, more efficient, and successful, then they might be characterized by an elevated level of work motivation. Such individuals might work more than the others to be more successful and to demonstrate their competence and knowledge in order to obtain more appreciation from important others (e.g., parents, partner, or manager). These associations have been supported by a recent study (i.e., Kun, Magi, et al., 2020; Kun, Urbán, et al., 2020) that emphasized the positive association between socially prescribed perfectionism and work addiction. These findings are in line with other studies examining the motivational background of work addiction (van Beek, Hu, Schaufeli, Taris & Schreurs, 2012; van Beek, Taris & Schaufeli, 2011). Individuals with work addiction show higher levels of introjected motivations which mean that they work more to avoid negative emotional states (e.g., anxiety, guilt, or shame). These negative emotions might be derived from socially prescribed perfectionism and the constant striving to be viewed as being good by important others.

According to the results of the present study, perfectionism is a more important risk factor for undergraduate students characterized by work addiction. This result may highlight the possible role of perfectionism as an antecedent of work addiction. Unfortunately, none of the existing longitudinal studies have examined the role of perfectionism for later work addiction, therefore in the future, it should be investigated.

When analyzing the association between self-esteem and work addiction, the overall correlation was not significant. In the previous meta-analysis by Clark and colleagues (2016), the same result was found. However, we also analyzed “global self-esteem” and “performance-based self-esteem” separately, because we assumed that these types of self-esteem factors have different connotations with work addiction. Our hypotheses were confirmed because global self-esteem showed a weak negative relationship with work addiction, while performance-based self-esteem correlated positively with work addiction. These results suggest that individuals characterized by work addiction have an intense desire to work to compensate for their low self-worth and they try to increase feelings of self-confidence by obsessive work (Burke, 1999, 2004; Killinger, 1991; Robinson, 1998a).

Although the present study does not answer questions about causes and consequences, it is presumed that a lower level of self-esteem increases motivation toward more intensive work. At the same time, the role of these people's performance in their self-evaluations is also important as earlier theories assumed (Fassel, 1990; Robinson, 1998a). If we have a look at performance-based self-esteem, we find that the basic internal motives toward overwork and self-imposed demands are not independent of performance (Machlowitz, 1980; Porter, 1996; Robinson, 1998a). Individuals with work addiction define themselves by their accomplishments and searched for success at work to feel more positive about themselves. Our results support the notion that those people whose self-esteem is highly based on their performance, excessive work can be an outstanding way to rate themselves better. Therefore, they will work more intensely and excessively to achieve more positive reinforcements which are crucial elements of their self-rating.

It was found that positive and negative affectivity were significant but weak positive correlates of work addiction. When analyzing the associations with the two constructs separately, positive affectivity as a personality trait did not correlate with work addiction. Therefore, we confirmed our hypothesis that work addiction does not relate to positive feelings. Consequently, describing individuals with work addiction as “happy people” or those who have a “positive addiction” can be a misinterpretation of the problem (Griffiths et al., 2018). On the other hand, the meta-analysis confirmed that negative affectivity showed a moderate positive relationship with work addiction. The type of the sample did not have an effect on negative affectivity because the same positive moderate correlation was found across all the analyzed samples. Several authors (e.g., Porter, 1996; Robinson, 1998a) have argued that individuals with work addiction may work to avoid negative feelings and the results here are in line with their hypothesis. Although negative affectivity moderately correlates with neuroticism (Levy, Cober, & Norris-Watts, 2003), these constructs are different. In a working context, negative affectivity has more of an effect on job satisfaction than neuroticism (Judge & Larsen, 2001), and Clark et al. (2010) found the similar results with work addiction (i.e., negative affectivity related to work addiction above the big five personality traits). The meta-analysis of the present study is in line with these results.

No overall association was found between neuroticism and work addiction (however, the positive correlation was significant among adult employees), but a higher level of negative affectivity was found to be a risk factor of work addiction. Only correlational results were included in the present study, so causal explanations between negative affectivity and work addiction cannot be confirmed. However, the results relate to two components of the addiction components model (Griffiths, 2005, 2011): mood modification and withdrawal symptoms. Negative affectivity (such as anger, anxiety, sadness, irritability, etc.) might lead to overwork as a mood modification strategy, and at the same time, individuals with work addiction might experience negative emotional states when they are unable to work (because they are ill, on holiday, etc.).

In the present study, the possible effects of gender distribution and the mean age recruited in the primary studies were also assessed. Neither gender nor age had any effect on the relationships between personality traits and work addiction. These findings are in line with earlier studies (e.g., Atroszko, Pallesen, Griffiths, & Andreassen, 2017; Burke & Matthiesen, 2004; Clark et al., 2016) which showed similarities in work addiction patterns among males and females and different age groups. Regarding the psychometric assessment of work addiction, we predicted that this variable would not moderate the relationship between personality and work addiction. On one hand, we confirmed that similar results were found for the different work addiction scales regarding self-esteem, perfectionism, positive affectivity, and negative affectivity. The results were mixed for the Big Five traits which appear to be more sensitive for the assessment of work addiction. On the other hand, we made only descriptive comparisons of correlations and did not compare the effect sizes statistically. Therefore, these results are not robust, and we regard them as only preliminary results of the moderator position of work addiction scales.

Among the personality factors assessed in the body of work addiction research, several dimensions were only assessed in one-one or two studies, and few of them (except type A behavior) were included in the previous meta-analyses (Clark et al., 2016; Patel et al., 2012). Our meta-analysis showed that narcissism, self-transcendence, trait anxiety, Type A behavior, obsessiveness, and persistence all have a positive correlation with work addiction. Since these results are based on only one or two studies, they should be interpreted them with caution and further studies are needed to confirm these preliminary results.

Both early theorists and authors of later models of work addiction have asserted that personality is a crucial element in work addiction (e.g., Liang & Chu, 2009). Ng et al. (2007) identified the key antecedents and consequences of work addiction and emphasized the importance of self-esteem, Type A personality, obsessive-compulsive personality, and need for achievement. According to the present results, it is clear that these individual factors are not enough to explain the risk of work addiction. To understand work addiction better, more studies are needed to assess the interaction between individual and environmental factors. For instance, Mazzetti, Schaufeli and Guglielmi (2014) tested the interaction between individual characteristics and workplace climate. They found that overwork climate facilitated work addiction especially among individuals high in perfectionism, self-efficacy, conscientiousness, and achievement motivation. Other authors (e.g., Griffiths & Karanika-Murray, 2012; Tóth-Király, Bőthe, & Orosz, 2018) have proposed integrative models of work addiction which take into account not just the micro-level characteristics (such as personality factors and other individual differences), but also meso-level (i.e. workplace climate, social norms, working conditions) and macro-level (i.e., culture, societal factors, economy) characteristics. Consequently, further research is needed to assess the possible interactions between these different levels of the antecedents.

In addition, research should focus not only on the interface between personality, societal, and environmental factors but on the interaction of traits, motivations, and cognitive aspects of personality. Motivations underlying work addiction are under-researched but it is assumed that both achievement motivations and work motivations can help to explain the pattern of work addiction better. For instance, Stoeber et al. (2013) confirmed that identified and introjected work motivations mediated the effect of perfectionism on work addiction. These findings are important and more studies on motivations are therefore needed. Another focus of future research should be longitudinal studies. Although a few follow-up studies have already been published (e.g., Andreassen, Bjorvatn et al., 2016; Falco et al., 2013; Shimazu, Schaufeli, Kubota, & Kawakami, 2012), the causal links between personality and other factors would be better explained through longitudinal studies.

### Limitations

The present study is characterized by some limitations. First, the systematic search found relatively few studies examining the relationship between personality and work addiction, especially when considering the different traits separately. Therefore, the statistical power of the main effects and the moderator analyses might have been low and thus non-significant results should be interpreted very carefully. Second, 82% of the studies included in the present review were conducted in Europe and the remaining 18% were conducted in North America. Consequently, these results represent the associations between personality and work addiction in the western world. Working habits, environmental, and other job-related factors may be different in other continents and there are likely to be societal and cultural factors underlying work addictions in other countries. Consequently, we cannot generalize the findings here to all the countries in the world. Third, work addiction (and other variables) was assessed by self-report scales. These instruments can assess the self-perception of individuals and how they rate themselves in the context of work. These ratings can be biased by positive self-presentation even though work addiction is not as stigmatized as other addictions (e.g., gambling disorder, alcohol use disorder, or other psychoactive substance use disorder). It would be interesting to have further data concerning the relatives, colleagues, and friends of individuals with work addiction, and how these individuals view their work-related behavior. To the present authors' knowledge, there are very few studies where such aspects have been reported (e.g. Bakker, Shimazu, Demerouti, Shimada, & Kawakami, 2014; Robinson, Flowers, & Ng, 2006). Fourth, the instruments used for assessing work addiction are suitable only for screening the risk of work addiction. These measures are not appropriate for making a clinical diagnosis of work addiction (especially as there are no official diagnostic criteria for work addiction). Fifth, only studies for the meta-analysis were selected where correlation coefficients were available. Although studies conducting ANOVAs, regression analyses, and path analyses report important results concerning the associations between personality and work addiction, they were not included in the meta-analysis. Sixth, as already mentioned, the present study is not suitable for testing causal relationships between personality factors and work addiction. Seventh, in relation to several personality traits or moderator factors, only one or two studies were included in the analyses. The results of these analyses should be interpreted very cautiously and these findings represent only tendencies between personality and work addiction and should be regarded as preliminary results in the field. Finally, all the studies (with one exception of one using random sampling) comprised convenience samples, therefore it is not clear whether results can be generalized to the general populations from which they were sampled. To our knowledge, there are only two countries where a nationally representative sample of the population was used to assess the risk of work addiction: Norway (Andreassen et al., 2014) and Hungary (Kun et al., 2020; Orosz, Dombi, Andreassen, Griffiths, & Demetrovics, 2016). However, only one of these (i.e., Andreassen et al., 2014) assessed the personality correlates of work addiction. In future research, there is a need to apply a more systematic sampling method, that is, consecutive sampling in clinical studies and population-based probability sampling methods in surveys.

## Conclusions

Despite the aforementioned limitations, the present meta-analysis has helped to clarify the role of personality factors underlying work addiction among the studies that have been carried out to date. It is concluded that personality appears to explain only a small part of the variance of work addiction. According to the meta-analysis presented here, perfectionism, global and performance-based self-esteem, and negative affect have the strongest and robust associations as personality risk factors of work addiction. Among the Big Five traits, a higher level of extraversion, conscientiousness, and intellect/imaginations contribute to an elevated risk of work addiction. It was also confirmed that gender and age are not relevant factors in the relationship between personality and work addiction. Although trait anxiety, obsessive-compulsiveness, and Type A personality showed a moderate positive correlation with work addiction, the empirical evidence regarding these dimensions is extremely limited. In future studies, it will be important to investigate these factors more extensively.

## Funding sources

Bernadette Kun was supported by the János Bolyai Research Scholarship of the Hungarian Academy of Sciences and by the ÚNKP-19-4 New National Excellence Program of the Ministry for Innovation and Technology. This study was supported by the Hungarian National Research, Development and Innovation Office (Grant numbers: FK134807, KKP126835, ELTE Thematic Excellence Programme 2020, KP2020-IKA-05). 

## Authors' contribution

BK, MJR, ZKT, and ZD contributed to concept and design. BK contributed to data collection. BK, MJR, and ZKT contributed to statistical analysis. BK and ZKT TK contributed to interpretation. MDG and ZD contributed to study supervision. All authors contributed to the writing of the original manuscript and the subsequent revised versions.

## Conflict of interest

ZD is the Editor-in-Chief of the Journal of Behavioral Addictions.

## Supplementary Material
