# Supplementary material for: Work addiction and personality: A meta-analytic study
Source: J Behav Addict. 2020 Dec 24;9(4):945–66. doi: 10.1556/2006.2020.00097 (PMC8969726; doi:10.1556/2006.2020.00097)
Supplement: Supplementary file 1 [file jba-9-945-s001.docx]

**Supplementary data**

**The effects of the type of sample on the relationship between work addiction and personality factors**

| **Outcome** | **Sample** | **N** | **K** | **r** | **CI 95%** | **Z** | ***P*** |
| --- | --- | --- | --- | --- | --- | --- | --- |
| Extraversion | Adult employees | 4315 | 5 | **0.042** | 0.012; 0.072 | 2.743 | .006 |
|  | Working students | 3147 | 3 | 0.048 | -0.007; 0.102 | 1.706 | .088 |
| Agreeableness | Adult employees | 3630 | 4 | 0.033 | -0.011; -0.076 | 1.473 | .141 |
|  | Working students | 3147 | 3 | -0.041 | -0.146; 0.065 | -0.762 | .446 |
| Neuroticism | Adult employees | 4610 | 6 | **0.188** | 0.097; 0.277 | 3.999 | <.001 |
|  | Working students | 3147 | 3 | -0.030 | -0.396; -0.167 | -0.167 | .868 |
| Conscientiousness | Adult employees | 4648 | 6 | -0.056 | -0.142; 0.031 | -1.253 | .210 |
|  | Working students | 3147 | 3 | **-0.080** | -0.114; -0.045 | -4.481 | <.001 |
| Openness | Adult employees | 516 | 1 | **0.201** | 0.117; 0.283 | 4.618 | <.001 |
|  | Working students | 3147 | 3 | 0.060 | -0.031; 0.150 | 1.297 | .195 |
| Perfectionism | Adult employees | 1865 | 5 | **0.315** | 0.248; 0.380 | 8.676 | <.001 |
|  | Undergraduate students | 325 | 1 | **0.520** | 0.436; 0.595 | 10.342 | <.001 |
|  | Working students | 456 | 2 | **0.118** | 0.068; 0.294 | 4.471 | <.001 |
| Negative affectivity | Adult employees | 1433 | 5 | **0.286** | 0.153; 0.410 | 4.110 | <.001 |
|  | Undergraduate students | 323 | 1 | **0.390** | 0.294; 0.478 | 7.389 | <.001 |
|  | Working students | 325 | 1 | **0.410** | 0.315; 0.497 | 7.792 | <.001 |
| Positive affectivity | Adult employees | 622 | 3 | -0.039 | -0.118; 0.040 | -0.975 | .329 |
|  | Undergraduate students | 325 | 1 | -0.040 | -0.148; 0.069 | -0.718 | .479 |
|  | Working students | 323 | 1 | 0.050 | -0.059; 0.158 | 0.895 | .371 |

Note: Bold text indicates a statistically significant correlation with a *p*-value less than 0.05.

**Moderating effects of the measurement instrument of work addiction**

| **Outcome** | **Scale** | **N** | **K** | **r** | **CI 95%** | **Z** | ***P*** |
| --- | --- | --- | --- | --- | --- | --- | --- |
| Extraversion | BWAS | 5938 | 5 | **0.038** | 0.013; 0.064 | 2.964 | 0.003 |
|  | DUWAS-10 | 1201 | 2 | 0.025 | -0.031; 0.082 | 0.882 | 0.378 |
|  | WC | 516 | 1 | **0.087** | 0.001; 0.172 | 1.976 | 0.048 |
|  | WE | 516 | 1 | 0.005 | -0.081; 0.091 | 0.113 | 0.910 |
|  | WART | 323 | 1 | **0.130** | 0.021; 0.236 | 2.339 | 0.019 |
| Agreeableness | BWAS | 5938 | 5 | -0.011 | 0.082; 0.059 | -0.314 | 0.753 |
|  | DUWAS-10 | 516 | 1 | 0.074 | -0.013; 0.159 | 1.670 | 0.095 |
|  | WC | 516 | 1 | **0.108** | 0.022; 0.193 | 2.456 | 0.014 |
|  | WE | 516 | 1 | 0.039 | -0.047; 0.125 | 0.884 | 0.377 |
|  | WART | 323 | 1 | 0.010 | -0.099; 0.119 | 0.179 | 0.858 |
| Neuroticism | BWAS | 5938 | 5 | **0.244** | 0.144; 0.338 | 4.724 | <.001 |
|  | DUWAS-10 | 1201 | 2 | 0.080 | -0.063; 0.218 | 1.098 | 0.272 |
|  | WC | 516 | 1 | **0.113** | 0.027; 0.197 | 2.570 | 0.010 |
|  | WE | 516 | 1 | **0.165** | 0.080; 0.248 | 3.772 | <.001 |
|  | WART | 323 | 1 | **-0.440** | 0.524; -0.348 | -8.448 | <.001 |
| Conscientiousness | BWAS | 5938 | 5 | **-0.115** | -0.147; -0.082 | -6.871 | <.001 |
|  | DUWAS | 333 | 1 | -0.070 | -0.176; 0.038 | -1.274 | 0.203 |
|  | DUWAS-10 | 1201 | 2 | **0.078** | 0.016; 0.139 | 2.473 | 0.013 |
|  | WC | 516 | 1 | **0.144** | 0.058; 0.227 | 3.284 | 0.001 |
|  | WE | 516 | 1 | 0.082 | -0.004; 0.167 | 1.861 | 0.063 |
|  | WART | 323 | 1 | 0.020 | -0.129; 0.089 | -0.358 | 0.720 |
| Openness | BWAS | 2824 | 2 | 0.024 | -0.064; 0.112 | 0.538 | 0.591 |
|  | DUWAS-10 | 516 | 1 | **0.201** | 0.117; 0.283 | 4.618 | <.001 |
|  | WC | 516 | 1 | **0.179** | 0.094; 0.261 | 4.098 | <.001 |
|  | WE | 516 | 1 | **0.223** | 0.139; 0.303 | 5.137 | <.001 |
|  | WART | 323 | 1 | **0.160** | 0.052; 0.265 | 2.887 | 0.004 |
| Global Self-esteem | BWAS | 378 | 1 | **-0.210** | -0.304; -0.111 | -4.128 | <.001 |
|  | WART | 809 | 2 | **-0.270** | -0.427; -0.096 | -3.007 | 0.003 |
|  | WAQ | 414 | 1 | **-0.300** | -0.385; -0.210 | -6.275 | <.001 |
| Performance-based self-esteem | DUWAS | 191 | 1 | **0.348** | 0.217; 0.467 | 4.77 | <.001 |
|  | WART | 3393 | 1 | **0.160** | 0.127; 0.193 | 9.397 | <.001 |
| Perfectionism | DUWAS | 1799 | 4 | **0.283** | 0.197; 0.364 | 6.253 | <.001 |
|  | WART | 997 | 3 | **0.347** | 0.132; 0.530 | 3.100 | 0.002 |
| Negative affectivity | DUWAS | 984 | 3 | **0.364** | 0.076; 0.595 | 2.452 | 0.014 |
|  | DUWAS-10 | 269 | 1 | 0.070 | -0.050; 0.188 | 1.144 | 0.253 |
|  | WC | 984 | 3 | **0.246** | 0.186; 0.304 | 7.828 | <.001 |
|  | WE | 984 | 3 | **0.282** | 0.217; 0.344 | 8.219 | <.001 |
|  | WART | 1459 | 4 | **0.400** | 0.333; 0.463 | 10.735 | <.001 |
| Positive affectivity | DUWAS | 253 | 2 | 0.000 | -0.105; 0.104 | -0.007 | 0.790 |
|  | DUWAS-10 | 269 | 1 | -0.090 | -0.207; 0.030 | -1.472 | 0.141 |
|  | WART | 648 | 2 | 0.005 | -0.083; 0.093 | 0.109 | 0.913 |

Note: Bold text indicates a statistically significant correlation with a *p*-value less than 0.05.
